# The epithelium in idiopathic pulmonary fibrosis: breaking the barrier

**DOI:** 10.3389/fphar.2013.00173

**Published:** 2014-01-10

**Authors:** Ana Camelo, Rebecca Dunmore, Matthew A. Sleeman, Deborah L. Clarke

**Affiliations:** Department of Respiratory, Inflammation and Autoimmunity, MedImmune LtdCambridge, UK

**Keywords:** epithelium, fibroblasts, idiopathic pulmonary fibrosis, apoptosis, TGF-β

## Abstract

Idiopathic pulmonary fibrosis is a progressive disease of unknown etiology characterized by a dysregulated wound healing response that leads to fatal accumulation of fibroblasts and extracellular matrix (ECM) in the lung, which compromises tissue architecture and lung function capacity. Injury to type II alveolar epithelial cells is thought to be the key event for the initiation of the disease, and so far both genetic factors, such as mutations in telomerase and MUC5B genes as well as environmental components, like cigarette smoking, exposure to asbestos and viral infections have been implicated as potential initiating triggers. The injured epithelium then enters a state of senescence-associated secretory phenotype whereby it produces both pro-inflammatory and pro-fibrotic factors that contribute to the wound healing process in the lung. Immune cells, like macrophages and neutrophils as well as activated myofibroblasts then perpetuate this cascade of epithelial cell apoptosis and proliferation by release of pro-fibrotic transforming growth factor beta and continuous deposition of ECM stiffens the basement membrane, altogether having a deleterious impact on epithelial cell function. In this review, we describe the role of the epithelium as both a physical and immunological barrier between environment and self in the homeostatic versus diseased lung and explore the potential mechanisms of epithelial cell injury and the impact of loss of epithelial cell permeability and function on cytokine production, inflammation, and myofibroblast activation in the fibrotic lung.

## INTRODUCTION

Idiopathic pulmonary fibrosis (IPF) is a devastating, fibroproliferative chronic lung disorder with complex and as yet unknown disease biology. The histopathology of IPF demonstrates a characteristic heterogeneity: areas of normal parenchyma interspersed with areas of paraseptal and subpleural fibrosis (The Idiopathic Pulmonary Fibrosis Clinical Research Network, 2012). At the cellular level, IPF is characterized by alveolar epithelial injury, initiation of inflammatory cascades, exaggerated pro-fibrotic cytokine expression, increased extracellular matrix (ECM) deposition, and the development of fibrotic lesions known as fibroblast “foci” (Wilson and Wynn, 2009). IPF has a heterogeneous clinical course, with a median survival after diagnosis of only 2.5–3.5 years (King et al., 2011). Although much of the pathogenesis is still to be elucidated, fibroblasts and epithelial cells, in particular type II alveolar epithelial cells and myofibroblasts are thought to be key drivers in the initiation and progression of the disease, respectively (Sakai and Tager, 2013). This review will focus on the epithelial cell, explore the mechanisms of cell injury and their role in repair in the fibrotic lung, as well as interactions with other key effector cells in IPF such as the fibroblasts.

## THE LUNG EPITHELIUM AS A PHYSICAL BARRIER AGAINST FOREIGN INSULTS

The airway epithelium is a pseudo-stratified mucosal barrier that consists of multiple cell types. It constitutes the first barrier of defense against environmental insults and infection by providing not only a mechanical and physical barrier to impede entry of foreign particles but also by its ability to orchestrate both the innate and adaptive immune responses. The lower airway surface, where gas exchange takes place is mainly covered by two types of alveolar epithelial cells, alveolar type I epithelial cells (ATI) that cover 90% of the airway surface due to their large flattened phenotype and whose main function is gas interchange, and alveolar type II epithelial cells (ATII) that are the most abundant epithelial cell type and whose function is to maintain the alveolar space by secretion of several types of surfactant proteins and other ECM components (Serrano-Mollar, 2012). The production of surfactant by ATII cells enables the gas exchange to occur by lowering the surface tension within the alveoli (Rackley and Stripp, 2012).

**FIGURE 1:**
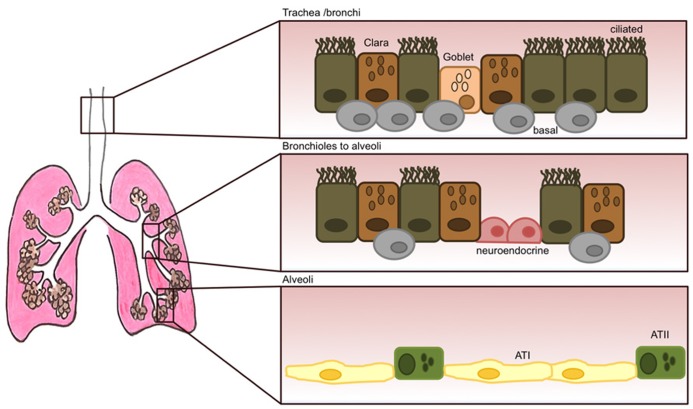
**The adult lung epithelium is composed of various different cell types.** The tracheo-bronchial epithelium forms a pseudostratified layer consisting of ciliated cells and secretory epithelial cells (Clara) and goblet cells. Underneath this layer, human basal cells (thought to be epithelial progenitor cells) are present at high numbers. Their numbers, however, decrease dramatically as the lung progresses into the alveolar space. Neuroendocrine cells can also be present that become innervated by ganglion cells. Their role is thought to be of regulating cell proliferation and differentiation. The respiratory bronchioles are still very poorly characterized and lead to the alveoli, that are mostly lined by alveolar type I (ATI) and type II (ATII) cells.

As well as alveolar epithelial cells in the alveolar compartment, other epithelial cell types populate the lung; with secretory Clara and goblet cells, ciliated, basal and neuroendocrine cells forming the tracheo-bronchial pseudostratified epithelium (**Figure 1**). Ciliated and secretory cells work in concert to clear the airway passages from micro-organisms, air pollutants and other inhaled pathogens. Mucous and goblet cells produce and release mucous into the apical surface of the epithelium thus trapping foreign particles (Roche et al., 1993) which are then cleared out by the action of ciliated cells beating in a rhythmic movement in the ascending direction (Fahy and Dickey, 2010; Gras et al., 2013). Mucous is a viscoelastic gel composed mainly of highly charged glycoproteins called mucins, and also some anti-viral and anti-inflammatory components such as lysozyme, defensins, IgA, and various cytokines (Nicholas et al., 2006). So far, 11 mucins have been identified in humans, with MUC5AC and MUC5B being predominant in human sputum (Rose et al., 2001). Mucin production importantly becomes up-regulated following viral infection to allow for better trapping and disposal of viral particles, however, over-production of mucous or over-proliferation of mucous-producing cells (goblet cell hyperplasia) can have deleterious effects by creating mucous plugs and thus leading to airway obstruction (Vareille et al., 2011). The latter is a common feature of several chronic lung diseases such as asthma, chronic obstructive pulmonary disease (COPD) and cystic fibrosis (Rogers, 2004; Hauber et al., 2006).

The epithelial integrity and permeability are maintained by tight connections within the epithelial cell layer, such as tight junctions, adherens junctions, and desmosomes. These cell–cell junctions provide strong adhesion, maintaining mechanical strength in the tissue, enabling communication between neighboring cells and blocking the entry of viruses, bacteria or inhaled allergens into the basolateral membrane where they can access epithelial cell receptors and/or activate antigen-presenting cells (Roche et al., 1993).

Mostly, the mature lung is non-proliferative, with prolonged survival of resident cells. However, in the event of damage to the epithelial layer following inflammation, infection, or exposure to airway pollutants, it is critical that the site of injury is quickly closed and replaced by newly differentiated epithelial cells in order to maintain an effective physical barrier. Several studies have identified some epithelial cell types and also progenitor cells in the lung as potential key regenerative players in the replacement of the epithelium after damage, although most of these remain relatively controversial. In the alveoli, ATII cells have been shown to proliferate and differentiate into type I cells (Cardoso and Whitsett, 2008; Ghosh et al., 2013) and in the conducting airways, basal cells (**Figure 1**), in contact with the basement membrane but not the airway lumen were found capable of long-term self-renewal and differentiation into ciliated and non-ciliated cell types *in vivo* (Hong et al., 2004). Lastly, neuroendocrine cells form clusters called neuroepithelial bodies, and there is some evidence these may play a role in regulating epithelial cell proliferation and differentiation of neighboring cells (Hoyt et al., 1991). The signaling and transcriptional programs that are activated in this process of wound healing can resemble and somewhat recapitulate early lung developmental programs (Rackley and Stripp, 2012). These pathways typically become dysregulated during chronic lung disease.

## THE EPITHELIUM IN INTERSTITIAL LUNG DISEASE

Alteration of the phenotype of alveolar epithelial cells is a central feature in IPF, whereby continuous damage to the epithelium and concomitant cell apoptosis are thought to contribute to the perpetuation of the fibrotic scarring (Jin and Dong, 2011). The causative event that initiates the fibrotic cascade in IPF is still unknown, although apoptosis or senescence of epithelial cells is arising as a hypothesis for the main initiator event (Chilosi et al., 2013). Indeed, recent studies found that IPF patients carry increased number of apoptotic cells in alveolar and bronchial epithelia (Plataki et al., 2005). The bleomycin mouse model supports this hypothesis by showing that inhibition of epithelial cell apoptosis prevents the development of the disease (Kuwano et al., 1999). This model is widely used in IPF research and shows the histological features of a fibrotic lung. It does, however, have limitations, as it is steroid responsive and the fibrosis resolves itself with time (Chandler, 1990), so it does not fully replicate the extent of the human disease.

What stimuli trigger the apoptotic cascade in epithelial cells is still under scrutiny. Cell senescence and premature aging due to genetic factors may be one cause but environmental factors such as cigarette smoking, viral infections, and gastroesophageal reflux (GER) are a few of the hypothesis that are currently being investigated.

Genetic mutations of telomerase, an enzyme that adds telomere repeats to the end of linear chromosomes, occur in 10% of familial IPF (Chilosi et al., 2012). Telomerase is known to maintain the precursor function in ATII cells and dysregulation of this enzyme greatly affects their regenerative capacity. Telomere shortening is dangerous for the cell as it causes DNA damage and induces cell death. Another disease-linked mutation that may lead to alveolar epithelial cell apoptosis occurs in the surfactant protein C gene which has also been found in familial IPF (Thomas et al., 2002). This mutation results in abnormal surfactant protein folding and accumulation of misfolded protein in the cell cytoplasm which activates the unfolded protein response (UPR) in an attempt to rescue the cell from cell death by halting the protein production. When this mechanism is not resolved, the cell enters a state of stress, called endoplasmic reticulum (ER) stress which ultimately leads to apoptosis (Noble et al., 2012). Other surfactant proteins, surfactant protein A and D have also been shown to be important mediators of respiratory infection susceptibility in mice (LeVine and Whitsett, 2001), which highlights the role of these proteins in the maintenance of the epithelial barrier. Environmental factors, like the mentioned viral infections but also cigarette smoking can induce UPR and ER stress, and in this way also contribute to accelerated telomere shortening and cellular senescence in the alveolar epithelia (Tsuji et al., 2004). Polymorphisms in the promoter region of the MUC5B gene have also been linked to IPF, this time not in ATII cells, but in bronchial epithelial cells (Seibold et al., 2011), suggesting that broader epithelial cell defects can affect the onset of disease. More recently, one study has found that MUC5B promoter polymorphisms were associated with interstitial lung disease in the general population, independently of cigarette smoking (Hunninghake et al., 2013). Despite this, in IPF patients, MUC5B polymorphisms actually associated with increase survival in another study (Peljto et al., 2013). These potentially contradicting reports underline the complexity of this disease, and prompt further, more functional studies to better understand the role of these genetic factors in inducing or driving disease pathology. A very recent study using genome-wide association (GWAS) has also identified 7 new susceptibility loci for pulmonary fibrosis associated with epithelial cell function, including DSP which encodes for desmoplakin, a component of the epithelial cell desmosome, and DDP9, also associated with epithelial cell adhesion and maintenance of its cytoskeleton (Fingerlin et al., 2013).

These studies are very important to identify genetic defects that underline the disease and not only help to increase our knowledge of the mechanisms that drive it but also to aid in finding potential therapeutic targets. This understanding may also lead to better methods of diagnostic, and increase the chances of an early diagnose that can better help patients. In fact, one study has looked at peripheral blood proteins and their association with disease and mortality and identified that high concentrations of matrix metalloproteinase 7 (MMP-7), interleukin (IL)-8, intercellular adhesion molecule 1 (ICAM-1), and vascular cell adhesion molecule 1 (VCAM-1) were associated with poor overall survival. Altogether, these proteins were shown to be involved in alveolar epithelial cell damage, oxidative stress, macrophage activation and neutrophil recruitment, all of which have been described in IPF (Richards et al., 2012). Lastly, a study of the transcriptional profiles of lung tissue from IPF patients has recently defined new genes associated with expression of cilium in epithelial cells which define particular subtypes of the disease, in this way helping in future patient stratification for therapies. In this study, patients with high cilium gene expression showed increased microscopic honeycombing, increased tissue expression of MUC5B and MMP-7, but with no effect on the number of fibroblastic foci, compared with other patients (Yang et al., 2013).

Genetic factors alone are not however the sole cause of IPF and it is well known that oxidative stress induced by chronic exposure to toxic substances, mainly cigarette smoke is a high risk factor for these patients. Cigarette smoke can induce oxidative stress and ultimately lead to cell death by causing an excessive production of reactive oxygen species (ROS), such as hydrogen peroxide, superoxide anions, and hydroxyl radicals that cells cannot scavenge and process (Psathakis et al., 2006). In IPF, the oxidative stress is continuously present as myofibroblasts that are constitutively activated by transforming growth factor beta (TGF-β) also produce high amounts of hydrogen peroxide. This has been elegantly demonstrated in an *in vitro* co-culture system whereby TGF-β-stimulated fibroblasts induced epithelial cell apoptosis through their production of hydrogen peroxide (Waghray et al., 2005). Exposure to cigarette smoke cannot, *per se*, induce IPF disease, and other chronic lung diseases such as COPD occur due to several years of cigarette smoke exposure. There are, however, differences between these two, not only different genetic susceptibilities but also the fact that the epithelial cell seem to be the main causative cell target in IPF, through senescence or apoptosis whereas in COPD, senescent markers have been more associated with mesenchymal cells like fibroblasts and endothelial cells (Chilosi et al., 2012, 2013).

**FIGURE 2:**
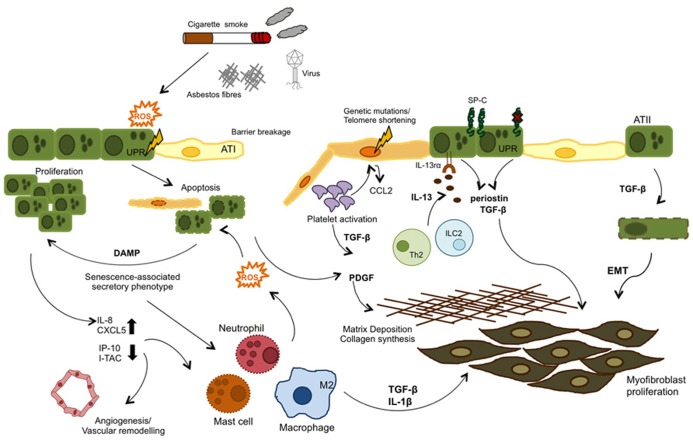
**Airway remodeling can be initiated by either aberrant epithelial cell (genetic mutations) or exposure to external irritants.** Genetic mutations in the telomerase enzyme and surfactant protein C (SP-C) are associated with a senescence phenotype in type II alveolar cells (ATII), Both these and reactive oxygen species (ROS) formed as a response to injury (cigarette smoke, asbestos, bleomycin, etc.) can activate the UPR response and induce ER stress and ultimately apoptosis in the epithelial cells. These events lead to a senescence- associated secretory phenotype, where injured epithelial cells release cytokines, chemokines, and danger-associated molecular patterns (DAMPs) which in turn recruit leukocytes to the site of injury and also activate the coagulation cascade, in an attempt to repair the damage. In IPF, these pathways become dysregulated and inflammatory cell types contribute to the unresolved damage/repair by secretion of TGF -β, IL -13, and ROS, which provide repeated injury to epithelial cells and induce their hyper-proliferation. TGF -β is also produced by activated platelets and contributes to the fibrotic state by inducing EMT, fibroblast proliferation and collagen synthesis, and ECM deposition.

Other potential initiators of lung fibrosis have been linked to viral infections, as these represent injury to the epithelium and lung and also activate the immune system (**Figure 2**). Some studies have found both Epstein–Barr virus (EBV) and herpes simplex virus (HSV) in alveolar epithelium of IPF patients, and that their presence was associated with poor prognosis (Tsukamoto et al., 2000; Margaritopoulos et al., 2013). Some recent theories arose that point to gastric contents as another potential cause of injury to the lungs in IPF, and GER has been associated with acute exacerbations in these patients (Lee et al., 2012). To support this observation an *in vitro* study has shown that a component of bile could induce TGF-β production by lung epithelial cells and also fibroblast proliferation, two key mechanistic features of IPF (Perng et al., 2007).

Once the epithelium is injured, the epithelial/fibroblastic pathways of wound healing become activated. The IPF lung shows a typical loss of integrity of the alveolar epithelium, with disruption of basement membrane integrity and collapse of the alveolar structure. The number of ATI cells is reduced whereas hyperplasia of ATII cells develops. These represent the progenitor cells that are able to differentiate to ATI cells and therefore re-epithelialize the site where the barrier was broken. However, the latter is seriously impaired in the IPF lung (Chapman, 2011). Other mechanisms of lung repair other than epithelial cell proliferation involve a migration of bone marrow-derived mesenchymal stem cells (BM-MSC) into the lung through chemokine gradients and their differentiation into epithelial cells. However, there are conflicting results on whether this process actually takes place in IPF (Rojas et al., 2005; Xu et al., 2009). Epithelial damage also activates fibroblasts to differentiate into myofibroblasts that then form the characteristic fibroblastic foci. The source of these myofibroblasts is still under debate but there are currently three hypotheses of how these cells are generated in the lung. The first one is the migration of local fibroblasts to the site of injury by expression of platelet-derived growth factor (PDGF) from platelets and TGF-β and tumor necrosis factor alpha (TNF-α) from the epithelium and their subsequent differentiation into myofibroblasts (Selman and Pardo, 2006). This hypothesis seems quite appealing due to the proximity of the cells to each other, and resting resident fibroblast can be immediately exposed to the cytokine and chemokine milieu secreted in response to damage and become activated. However, this may account for the initial burst of the fibrotic response but may not be sufficient in the full blown disease, where myofibroblast take over large sections of the lungs. The second theory proposes that myofibroblasts may be derived from circulating CXCR4-positive fibrocytes (or circulating mesenchymal cells), and that these are attracted to the lung by the high expression of chemokine CXCL12 from epithelial cells (Andersson-Sjoland et al., 2008; Antoniou et al., 2010). In favor of this hypothesis, a higher number of circulating fibrocytes have been found in the blood of IPF patients compared with healthy controls (Andersson-Sjoland et al., 2008). The third hypothesis has been given a lot of attention recently and is thought to occur through a loss of the characteristic epithelial cell phenotype, such as E-cadherin expression and the de-differentiation of the epithelial cell into a myofibroblast by gaining of mesenchymal markers like α-smooth muscle actin and fibronectin, in a process called epithelial-to-mesenchymal transition (EMT; Willis et al., 2006). TGF-β, the main cytokine regulating fibrosis, is thought to drive EMT directly and perpetuate this event in the lung (Guarino et al., 2009). A key pathway that has been found dysregulated in fibrosis involved in EMT is the Wnt-signaling pathway. Hyperplastic ATII cells overexpressing the WNT-1 inducible signaling protein or WISP-1 up-regulate the secretion of pro-fibrotic markers like MMP-7 and plasminogen-activator inhibitor 1 (PAI-1) which could induce EMT in the neighboring epithelium (Margaritopoulos et al., 2013).The Wnt pathway has been shown to be activated by cell senescence, which triggers a “senescence-associated secretory phenotype” whereby injury-induced apoptosis in epithelial cells initiates the release of damage factors or alarmins that target the neighburing type II alveolar epithelial cells to induce their proliferation in an attempt to restore homeostasis (Chilosi et al., 2012).

In addition to above mentioned genetic and environmental factors that can induce epithelial cell damage in fibrosis and the mechanisms that it activates (apoptosis, cell proliferation, release of pro-inflammatory, and pro-fibrotic cytokines) which perpetuate the damage/repair response, the mechanical stress to the lung is another cofactor in inducing alveolar damage. The mechanical stretch focused on limited parts of lung parenchyma has detrimental effects in alveolar epithelial permeability and tissue regeneration following injury and has also been showed to increase the production of ROS (Chilosi et al., 2013; Davidovich et al., 2013). In fact, inducing mechanical stretch and compression in *in vitro* cultures of epithelial cells inhibited wound closure by inhibiting both cell spreading and cell migration and this was dependent on the duration of the stretch cycles (Savla and Waters, 1998). Moreover, αvβ6-mediated activation of TGF-β has been shown to require cellular tension and thus increased stiffness of the lung tissue may form a positive feedback loop promoting progression of the fibrosis (Giacomini et al., 2012).

## THE INJURED EPITHELIUM AND THE IMMUNOLOGICAL RESPONSE

Despite immune suppressors, such as steroids having had little to no effect in the clinic for the treatment of IPF, injury to the epithelium typically elicits an immune response in the lung. The coagulation cascade is the first mechanism activated in the wound-healing process, and activated platelets release pro-fibrotic factors like PDGF and TGF-β1 (Chambers, 2008). Damaged epithelial cells release a variety of the chemokines that recruit inflammatory monocytes and neutrophils to the site of injury. In a single injury, such as infection or allergen exposure, monocytes differentiate into phagocytic macrophages that phagocytose the fibrin clot and neutrophils remove debris and kill invading bacteria. In the case of repeated injury such as the one occurring in COPD and IPF, neutrophils and macrophages are not eliminated quickly enough and their presence can further exacerbate the fibrotic cascade by continuous production of ROS (Wynn and Ramalingam, 2012). The recruitment of neutrophils to the bronchoalveolar space is considered a predictor of early mortality in IPF patients (Kinder et al., 2008), and both macrophages and neutrophils have been identified as pro-fibrotic cell types in mouse models of pulmonary fibrosis (Pardo et al., 2000; Duffield et al., 2005). Other innate myeloid cell types that have also been suggested as having a pro-fibrotic role in the lung include eosinophils and mast cells (Wynn and Ramalingam, 2012) and, more recently a newly identified cell type, innate lymphoid cells 2 (ILC2) has also been implicated as a mediator of hepatic fibrosis (McHedlidze et al., 2013). Within the adaptive immune system, there is some evidence that CD4^+^ Th1, Th2, and Th17 subtypes may play role in pulmonary fibrosis and their plethora of cytokines such as interferon gamma (IFN-γ) for Th1 and IL-4 and IL-13 for Th2 cells have been linked to disease development in the lung (Wynn, 2011). Interestingly, IPF fibroblasts are hyperresponsive to IL-13, and increased expression of IL-13 and its receptor IL-13R-α1 correlate with disease severity (Murray et al., 2008). As well as its direct effects on fibroblast proliferation and epithelial cell apoptosis (Borowski et al., 2008), IL-13 can also target TGF-β directly *in vivo* which further augments the fibrotic response (Lee et al., 2001). The strategy of targeting IL-13 in IPF is currently undergoing clinical trials. Regulatory T cells have also been associated with IPF, however, there is still some controversy to whether their role in IPF is pro- or anti-fibrotic (Kotsianidis et al., 2009; Liu et al., 2010).

Toll-like receptor (TLR) activation in epithelial cells by could be the trigger responsible for the epithelial-induced immune cell recruitment to the lungs and TLR signaling pathways have also been linked with tissue repair, as they can promote tissue remodeling (Jiang et al., 2005). In line with the hypothesis of viral infections as one of the insults that could initiate IPF, TLR9 (that recognizes nuclei acid strands) has been found overexpressed in fibroblast of IPF patients (Margaritopoulos et al., 2010). In a more recent study, TLR2 was also found up-regulated in the lungs of IPF patients compared with healthy controls (Samara et al., 2012). This receptor has also been found to be critical for the release of pro-inflammatory cytokines and promotion of collagen and fibronectin deposition following bleomycin exposure (Razonable et al., 2006; Yang et al., 2009). In addition, in a radiation-induced lung fibrosis model, both TLR2 and TLR4 were found to have a protective effect by preventing epithelial cell injury and suppressing fibrogenesis (Paun et al., 2010). Activation of TLR2 and TLR3 can also induce up-regulation of mucin expression (Li et al., 1997; Chen et al., 2004), and therefore a dysregulation of TLR signaling can impact not only the type of immune response initiated but also the balance of mucous production and clearance. This can have a major impact during viral exacerbations in IPF patients, which are one of the major causes of mortality in these patients. A recent study places TLR3 as an important factor in IPF. It was found that in IPF-derived fibroblasts carrying the TLR3 L412F polymorphism, TLR3 activation resulted in abnormal cytokine production. Moreover, TLR3-deficient mice showed increased collagen production in the lungs following bleomycin-induced fibrosis and patients carrying this polymorphism had significantly greater risk of mortality and accelerated decline in forced vital capacity (O’Dwyer et al., 2013).

Though well-known sentinels for the recognition of pathogen specific patterns, TLRs have also been shown to identify some endogenous ligands, including fragmented forms of hyaluronic acid (a major component of ECM in most organs) which could signal through TLR2 and TLR4 receptors in immune cells (McKee et al., 1996; Scheibner et al., 2006). Other endogenous TLR ligands reported include HMGB1, signaling through TLR2 and TLR4. HMGB1 is a nuclear protein that can either be released by either activated immune cells following inflammation or by necrotic cells (Scaffidi et al., 2002). More recently, fibrinogen cleavage products have also been found to act as TLR4 ligands in both alveolar macrophages and epithelial cells, and that this interaction up-regulated the gene expression of IL-13Rα1 and MUC5AC in both these cell types (Millien et al., 2013). These endogenous patterns are usually referred to as danger-associated molecular patterns (DAMPs) and it can be easily hypothesized that they may be of importance in chronic lung diseases where the epithelial barrier is severely damaged, although further studies will be needed to identify the extent to which they may either cause it or contribute to their perpetuation.

An autoimmune response has been also suggested as part of the IPF pathology, where B cell aggregates were found in patient’s lungs and circulating activated CD4 T cells in their serum (Marchal-Somme et al., 2006). Indeed some studies have found circulating auto-antibodies against epithelial antigens in the serum of IPF patients, including antibodies against cytokeratins and, more recently an anti-periplakin antibody (Fahim et al., 2012). The latter was shown to delay wound repair *in vitro*, by decreasing epithelial cell migration (Taille et al., 2011). Therefore, there is some evidence of a chronic inflammatory setting in IPF, however, further investigation is warranted to understand whether this is caused by the aberrant wound-healing and permanent secretion of pro-inflammatory modulators by epithelial cells and fibroblasts, or whether the initial lung injury provokes a deregulated immune response and the immune cell-derived cytokines perpetuate the aberrant apoptosis/cell proliferation in epithelial cells and the continuous differentiation and proliferation of myofibroblasts.

## TGF-β AS A MOLECULAR MEDIATOR OF IPF

TGF-β is a member of the TGF-β superfamily and exists in three different forms in mammals which are expressed throughout the body (TGF-β1, TGF-β2, and TGF-β3) (Moustakas and Heldin, 2009). TGF-β signaling is important for morphogenesis during embryonic development and in adulthood, for tissue homeostasis, however, in injured tissue, TGF-β acts as a major pro-fibrotic cytokine, inducing fibroblast recruitment, proliferation and differentiation of myofibroblasts, and deposition of ECM (Sheppard, 2006). The role of TGF-β is therefore well described in IPF; it is overexpressed in both patients (Coker et al., 2001) and animal models of lung fibrosis and blocking TGF-β signaling improves pulmonary fibrosis in mouse models (Bonniaud et al., 2005). TGF-β1 is produced as a complex of active TGF-β1 non-covalently associated with the latency-associated peptide (LAP) and these complexes are sequestered in the ECM. In order to exert its biological function TGF-β must be released from these complexes. This process can be caused by physical mechanisms such acidification, and oxidation, which could be initiated by injury, for example, from asbestos exposure (Sullivan et al., 2008). TGF-β can also be cleaved by proteases such as MMP-2 and MMP-9 (Jenkins, 2008). More relevant to the epithelium is that epithelial cell integrins, more specifically αvβ6 can induce conformational changes in the latent TGF-β complexes, and this process has been found to be a key event in pulmonary fibrosis (Tatler and Jenkins, 2012). The role of integrins will be further explored in the next section of this review. Once released from the latent complexes, TGF-β can interact with its receptors on the surface of fibroblasts, and in the canonical signaling pathway it phosphorylates Smad proteins (Smad2 and Smad3) which then heterodimerise with Smad4 to form Smad2/4 and Smad3/4 complexes that translocate to the nucleus and bind to the promoter regions of pro-fibrotic genes like collagen, fibronectin, and α-smooth muscle actin (Santibanez et al., 2011). In the non-canonical activation pathways, the c-Abelson tyrosine kinase (c-Abl) and mitogen-activated protein kinase (MAPKs) have been shown to be directly activated by TGF-β (Daniels et al., 2004; Hu et al., 2006). In contrast to the effects of protecting fibroblasts from apoptosis and inducing their differentiation, TGF-β promotes apoptosis in lung epithelial cells, and this has been shown both *in vitro* (Solovyan and Keski-Oja, 2006) and in a mouse model where protection from bleomycin-induced fibrosis was achieved in animals harboring a epithelial-specific deletion of the TGF-β type II receptor (Li et al., 2011). This way TGF-β is a bidirectional modulator, affecting both epithelial cells and fibroblasts in IPF.

## EPITHELIAL CELL INTEGRINS AND CHEMOKINES

**FIGURE 3:**
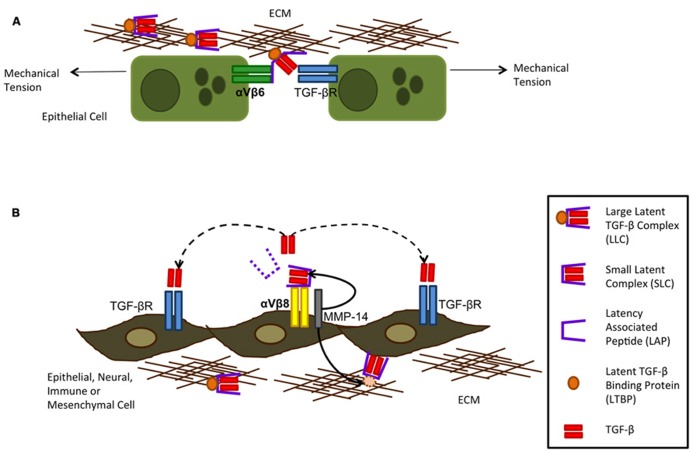
**Integrin activation of TGF-β can occur via αVβ6 (A) and αVβ8 **(B)**.** Integrin αVβ6 is restricted to the epithelium and can activate TGF -β following mechanical tension of the cells alongside binding of the large latent complex (LLC) to αVβ6 and extracellular matrix (ECM) proteins. This tension exposes the active TGF -β through opening of the latency-associated peptide (LAP) allowing it to bind to its receptor on neighboring epithelial cells only as TGF -β is not released from the cell surface. Integrin αVβ8 requires metalloproteolytic cleavage by MMP-14 which results in TGF -β being liberated from its latent complex and therefore acting on cells by paracrine diffusion. Although integrin αVβ8 is more widely expressed on several subsets of cells, metalloprotease expression is required for it to activate TGF -β (figure adapted from Nishimura, 2009).

Integrins are heterodimeric transmembrane receptors made up of one α and one β subunit which bind to ECM proteins. So far 26 combinations have been identified. They function by transducing information between the ECM and the inside of the cell and have important roles in adhesion, migration, survival, differentiation, invasion, and maintenance of cell shape. *In vitro*, αVβ3, αVβ5, αVβ8, and αVβ6 can all activate TGF-β1 and TGF-β2 through binding of the arginine–glycine–aspartate (RGD) motif which is present in the LAP of the latent complex of TGF-β, although αVβ6 and αVβ8 are higher affinity binders. *In vivo*, however, the role for αVβ6 and αVβ8 in TGF-β activation is still under investigation, although expression of αVβ3 and αVβ5 is up-regulated in systemic sclerosis and αVβ5 is detected in the fibroblast foci of patients with IPF (Asano et al., 2005). Upon binding of LAP to αVβ6 or αVβ8, cleavage or conformational change occurs, allowing the active TGF-β to interact with its receptor (**Figure 3**). The mechanisms by which αVβ6 and αVβ8 activate TGF-β are different however. For αVβ6, mechanical stretching of the cells results in a conformational change of the latent complex revealing the active TGF-β peptide and allowing it to bind to its receptor on cells in direct contact with each other. On the other hand, αVβ8 requires proteolytic cleavage with the help of MMP-14 releasing the active TGF-β peptide from the latent complex and allowing it to interact with its receptor in a paracrine fashion (Annes et al., 2003). The integrin αVβ8 has been shown to be expressed on several cell types such as epithelial, fibroblast, neural, and immune whereas αVβ6 is predominantly expressed on epithelial cells (Munger et al., 1999). Under normal conditions αVβ6 is expressed at low levels and further induced by inflammatory mediators unlike αVβ8 which it is expressed at high levels in the normal airway epithelium. This suggests αVβ6 may be more predominant at TGF-β activation during epithelial injury. During epithelial injury, damage to the basement membrane further allows αVβ6-expressing epithelial cells to interact with other cell types such as mesenchymal cells allowing TGF-β to exert fibrotic effects upon other cells.

In mice that do not express the β6 integrin subunit there is a decrease in active TGF-β in the lung (Jenkins et al., 2006) and these mice can develop MMP-12-dependent emphysema suggesting epithelial specific activation of TGF-β *in vivo* is important. In addition blockade of αVβ6 either in knockout mice or through antibody neutralization protects mice from bleomycin induced fibrosis emphasizing its importance in fibrogenesis. Furthermore patients diagnosed with lung fibrosis also show enhanced levels of αVβ6 and currently there is an anti- αVβ6 (STX-100) from Stromedix in clinical development for organ fibrosis.

Angiogenesis occurs as part of the wound healing process and it has been described around the fibroblastic foci in both IPF patients and in the bleomycin model (Cui et al., 2001; Sterclova et al., 2009). CXC chemokines are important factors in the angiogenic–angiostatic balance as well as being powerful chemoattractants for inflammatory cells (Gerard and Rollins, 2001). These can be further divided into two subgroups: ELR + CXC (pro-angiogenic) binding to CXCR2 or ELR–CXC (anti-angiogenic) biding to CXCR3, depending on the presence or absence of a glutamate–leucine–arginine (ELR) motif, respectively (Strieter et al., 1995). To date, several CXC chemokines have been implicated in IPF (**Figure 2**). IL-8 (or CXCL8), a potent neutrophil chemoattractant produced by epithelial cells, endothelial cells, and macrophages has been shown elevated in BAL fluid, lung tissue, and serum of IPF patients (Prasse and Muller-Quernheim, 2009). Recently, another epithelial-derived chemokine, CXCL6 (or GPC-2) was shown to be elevated in BAL fluid of both IPF patients and in the bleomycin model and that therapeutic blockade of this chemokine in this model significantly decreased both inflammation and some fibrosis markers (Besnard et al., 2013). In contrast, angiostatic CXC-chemokines such as CXCL10/IP-10 and CXCL11/I-TAC were found to de decreased in IPF patients (Sterclova et al., 2009). Apart from these, CC chemokines such as CCL2/MCP-1 has also been shown as important effectors in IPF pathogenesis by recruiting macrophages to the lung (Agostini and Gurrieri, 2006). Moreover CCL2 has been recently found to be expressed in airway epithelial cells (Mercer et al., 2009).

Two studies have found that IFN-γ-1b therapy could have beneficial effects in IPF by regulating the angiogenic balance in IPF. One study found that subcutaneous IFN-γ-1b delivery three times weekly for 6 months, increased CXCL11/I-TAC in both BAL fluid and plasma and that CXCL5 and type I collagen were significantly reduced (Strieter et al., 2004). The second study showed that several pro-angiogenic chemokines such as IL-8 and CXCL5 were up-regulated following IFN-γ-1b treatment but no changes were seen in the levels of IP-10 or I-TAC (Antoniou et al., 2008). Moreover, they did not see any correlation with improvement in lung physiology or disease outcome. Therefore further research is needed to fully understand the potential benefits of this drug.

## SUMMARY AND FUTURE DIRECTIONS

Idiopathic pulmonary fibrosis is a complex disease that is refractory to treatment and carries a high mortality rate. The pharmaceutical and biotechnology industry have made many attempts to find effective treatments for IPF, but the disease has so far defied all attempts at therapeutic intervention. Clinical trial failures may arise for many reasons, including disease heterogeneity, lack of readily measurable clinical end points other than overall survival, and, perhaps most of all, a lack of understanding of the underlying molecular mechanisms of the progression of IPF. The anti-fibrotic drug Esbriet (pirfenidone) is the only drug marketed for IPF, however, it is not yet approved in the US. Other promising candidates in clinical development include the tyrosine kinase inhibitor nintedanib in Phase III (Boehringer Ingelheim), the monoclonal antibodies tralokinumab (MedImmune: AstraZeneca) and lebrikizumab (GenenTech) targeting anti-IL-13, the monoclonal antibody STX-100 against integrin αVβ6 (Biogen Idec), the lysophosphatidic acid 1 inhibitor (Bristol-Myers Squibb) and the monoclonal antibody simtuzumab against lysyl oxidase-like 2 (Gilead) in Phase II.

Other trials targeting the immune system, using steroids as immune suppressants have had less success, and in fact have been associated with deleterious effects in IPF (The Idiopathic Pulmonary Fibrosis Clinical Research Network, 2012). However, despite current advances in technology and other current therapies in the clinic, lung transplantation is the only current available treatment for IPF that has been shown to improve survival (Adamali and Maher, 2012), but it carries the downside risks of donor availability, infection, and organ rejection.

A deeper understanding of the mechanisms that initiate the fibrotic pathway is urgently needed in order to develop more appropriate and more specific therapies. Accumulating evidence suggests that alveolar epithelial cell apoptosis may be the initial trigger of the disease and both genetic background and environmental exposure contribute to this outcome and initiate the “senescence-associated secretory pathway” that ultimately leads to full blown disease. It is imperative to find the common ancestor and disease initiator, so that patients can be identified earlier in the clinic and perhaps have more adequate treatment. Since this is a complex disease involving multiple pathways, it is possible that the future best therapies will involve combination drugs that target more than one pathway.

## AUTHOR CONTRIBUTIONS

Ana Camelo as first author led the direction and wrote a large proportion of the review, with input from both Deborah L. Clarke and Rebecca Dunmore. Matthew A. Sleeman provided direction and guidance.

## Conflict of Interest Statement

The authors declare that the research was conducted in the absence of any commercial or financial relationships that could be construed as a potential conflict of interest. The authors and editor declare that while they are currently employed by the same institution there was no conflict of interest during the review and handling of this manuscript.
